# Refractory Methamphetamine-Induced Psychosis: An Emerging Crisis in Rural America and the Role of Amantadine in Therapeutics

**DOI:** 10.7759/cureus.22871

**Published:** 2022-03-05

**Authors:** Mayank Gupta, Nihit Gupta, Michael Esang, Angelica Antai, Jeffrey Moll

**Affiliations:** 1 Psychiatry, Lake Erie College of Osteopathic Medicine, Erie, USA; 2 Psychiatry and Behavioral Sciences, Clarion Psychiatric Center, Clarion, USA; 3 Psychiatry, University of West Virginia, Glen Dale, USA; 4 Psychiatry and Behavioral Sciences, Nassau University Medical Center, East Meadow, USA; 5 Radiology, University of Uyo Teaching Hospital, Uyo, NGA

**Keywords:** rural, amantadine, psychosis, methamphetamine, meth

## Abstract

In the last decade, methamphetamine (MA) use has substantially increased in rural America. These changes in the epidemiological trends could be attributed to the restricted availability of opioids after measures against the opioid epidemic were enforced. The availability of cheaper alternatives, such as fake prescriptions mixed with fentanyl, is a few among the many recent developments in the ongoing mental health and substance abuse crisis in rural America. A serious clinical effect of MA use is psychosis, which inadvertently has stretched mental health services. In recent times, the atypical clinical presentations of these psychotic episodes with a refractory course have challenged clinicians. Hence, the knowledge of its unique pharmacodynamics, neurotoxicity, similarities with schizophrenia amid the evolving empirical evidence is critical to addressing this unique conundrum.

## Introduction

Stimulant use disorder affects between 0.3% and 1.1% of the population and costs more than $85 billion per year globally. Methamphetamine (MA) and its derivative, 3,4-methylenedioxymethamphetamine (MDMA), are extensively abused drugs, with serious acute and long-term effects. MA-related psychosis (MAP) has been discussed in the literature since the last century and is associated with a conventional and prevailing notion of self-limiting illness after the cessation of substance use. In the last two decades and especially after the crackdown following the opioid epidemic affecting rural America, the incidence of MA use has increased exponentially.

In 2005, The Combat Methamphetamine Epidemic Act was passed as an essential measure to curb the availability of pseudoephedrine, which is an important ingredient for manufacturing MA [1]. However, to circumvent these measures, manufacturers in Mexico are using phenyl-2-propanone (P2P), a precursor of pseudoephedrine. In 2021, two more alarming trends were observed. Firstly, the American Medical Association reported that even though the rates of opioid prescriptions dropped by 44% in the last decade, the number of deaths continued to increase (about a 30% increase in 2020). Secondly, the Drug Enforcement Administration issued its first safety alert in six years, about the alarming increase in the fake prescription pills containing fentanyl and MA contributing to high mortality rates. The global epidemiological trends point towards increased MA use and the prevalence of recreational use varies as per demographics, but the risk of psychosis among MA users is two to three times higher in comparison to non-users. The risk is even higher when the use starts at a younger age, when used in larger amounts, and when used in the crystallized form. There is a dose-dependent relationship involving the modes of use, frequency, and amount, and prolonged use significantly increases the odds of psychosis [2].

These changes in epidemiolocal trends could be attributed to increased availability, lowered risk perception, access to cheaper alternatives to cocaine and opiates, and higher potency of the drugs with more addictive potential. Many high-risk groups exist, but increased use among pregnant women is of serious concern given its neurodevelopmental effects and risk of neonatal abstinence syndrome in infants. MA is a potent CNS stimulator that competitively inhibits dopamine re-uptake at the dopamine transporter (DAT) and increases DAT-mediated reverse-transport of dopamine from the cytoplasm into the synaptic cleft independent of action potential-evoked vesicular release. Interestingly, MA acts primarily as a DAT blocker at low concentrations and reverses dopamine transport at high concentrations [3]. Animal studies suggest that excess dopamine or glutamate contributes to neuroinflammation, and apoptosis in MA-induced neurotoxicity, and that protein kinase Cδ might mediate these effects. Also, MA-induced dysfunction in the dopamine D1 receptor-extracellular signal-regulated kinase 1/2 (ERK1/2) pathway in the prefrontal cortex has been associated with the effects on long-term memory [4]. An MA user having lower dopamine D2/3 receptor availability at baseline could be a predictor of relapse following treatment.

There are few differences between MAP and primary psychotic disorders like schizophrenia; however, a preponderance of tactile hallucinations and formication is typical of MAP. In the last few years, complex clinical presentations of MAP have challenged mental health professionals and raised some serious questions. Firstly, why is there a recent surge in MAP-related psychiatric admissions in the absence of any epidemiological data that reflects these trends? Secondly, is the increase in MA use related to policies implemented to address the opioid crisis? Thirdly, are there any changes in the potency or pharmacodynamics profiles that are associated with more severe lasting effects? And lastly, is there a consensus on treatment strategies for refractory MAP amidst this emerging crisis?

In light of the factors discussed above, we present a clinical case to underscore these issues and provide a succinct overview of approaches toward managing MAP.

## Case presentation

A 30-year-old single, unemployed, male patient was admitted with florid psychotic symptoms. During his initial clinical assessment in the ER, he believed his mind had been “taken” and was responding to hallucinations. During his previous hospitalizations, he had been given a diagnosis of bipolar II disorder; he also had an extensive history of a substance use disorder, including MA, opioids, and cannabis. He was on Suboxone for opioid use disorder maintenance treatment, and medical marijuana, and was not compliant with his prescribed olanzapine, mirtazapine, and buspirone for an indeterminate period. He had a legal history with three counts of drug-related charges and probation violations.

At the time of his admission, he was exhibiting highly disorganized behavior, was unable to sit still, and displayed rapid and pressured speech. His thoughts were tangential, with loosening of association, persecutory, somatic delusions, and second-person auditory hallucinations. His long-term memory had been impaired. His urine was positive for MA and cannabinoids but was notably negative for buprenorphine. Liver enzymes were mildly elevated. A CT brain was done, which was negative for any acute intracranial pathology. His family provided the collateral history and reported extensive use of MA in the last six months. He was started on olanzapine 5 mg two times a day and oral lithium 600 mg once a day. His inpatient hospital course, however, was marked by resistance to multiple antipsychotic medications with ongoing agitated and aggressive behaviors. Persecutory delusions and hallucinations persisted, and his aggressive behavior necessitated the use of psychotropics as needed with minimal response. The following medications were tried in this patient to address psychosis and agitation: olanzapine, fluphenazine, clonazepam, haloperidol, lorazepam, risperidone, quetiapine, asenapine, lithium, sodium valproate, chlorpromazine, and propranolol. However, none of them was effective either with single-use or in combinations. After conducting a clinical case discussion with peers, the decision was made to initiate clozapine. Clozapine was slowly titrated to 200 mg once a day. The patient’s intrusive, aggressive, and agitated behavior began to remit. Although his behavior and symptoms improved, he remained intermittently aggressive, agitated, with persecutory delusions. Amantadine 100 mg twice daily was subsequently added to his pharmacologic regimen and this yielded further improvement in his symptoms. He became less agitated and paranoid. He continued to progress in his clinical recovery and was eventually transitioned to step-down care for continued management of his residual symptoms.

## Discussion

It has been known that symptoms of MAP usually remit within a few weeks to a month after the cessation of MA use. However, recent studies have shown that in 30% of patients, symptoms of MAP may persist for up to six months after cessation, and in another 10-28%, they persisted for more than six months after quitting [5]. There is growing empirical evidence to support considering MAP as a distinct entity different from schizophrenia. Although the verdict is still out, the increased risk of MAP in an individual with a family history of schizophrenia and the role of epigenetics need to be further examined [6]. However, if the symptoms persist beyond six months, as per The Diagnostic and Statistical Manual of Mental Disorders, Fifth Edition (DSM-5), a diagnosis of a primary psychotic disorder with a specifier would be more appropriate for accurately recording the epidemiological data. And after ruling out cannabis-induced psychosis, hallucinogen persisting perceptual disorder needs to be considered as well.

It has been proposed that excess cortical glutamate activity leading to GABAergic interneuron damage underlies the pathophysiology of MAP. The animal studies-based theory behind cortical GABAergic interneuron's vulnerability to glutamate overactivity points to the subcellular location of N-methyl-D-aspartate (NMDA) receptors on interneurons in the cortex. Neuroimaging studies in human subjects, however, appear to lend weight to these assertions. Although the mechanism is unknown, Hsieh et al. identified the hippocampus, anterior cingulate, caudate, nucleus accumbens (NAc), thalamus, and the amygdala as pertinent sites in the brain involved or affected in the pathophysiology of MA neurotoxicity, and potentially MAP. They argued, again based on animal models, that the steady glutamate overflow from the thalamus and NAc to the cortex due to overexposure to MA may lead to damage to the GABAergic interneurons, which in turn would result in the dysregulation in glutamate signaling in the cortex and MAP. The multi-modal brain imaging techniques involving PET scans were able to highlight decreased glucose metabolism among MA users compared to healthy controls (HCs) in the left insula, left precentral gyrus, and the anterior cingulate cortex [7]. The glucose metabolism and cerebral perfusion in the frontal, striatal, and limbic regions are disrupted in individuals with MAP. Significantly decreased regional glucose metabolism in MA users (individuals with MA use disorder and those with MAP) compared to HCs has been observed. The statistical map was thresholded by using an uncorrected p-value <0.001 at the voxel level combined with a family-wise error (FWE)-corrected p-value <0.05 at the cluster level [7]. Table 1 provides a summary of epidemiological trends and risks factors associated with MAP.

**Table 1:** Unique aspects of MAP highlighted by recent empirical research

Methamphetamine-related psychosis (MAP)
National Survey on Drug Use and Health (NSDUH)'s estimate of the lifetime prevalence of MA use was about 5.6% in 2020
Chronic MA users have five times higher risk of psychosis
Younger age, mode, and larger quantities are attributable to higher risk
Neurobiology of MA use-related neurotoxicity is distinct from schizophrenia
10-28% of treatment-refractory MAP symptoms persist for more than six months after cessation
Polysubstance use is common among MA users and fake prescriptions combinations are associated with higher mortality

Neurotoxicity with chronic MA may lead to brain volume reduction in the affected regions. Previous studies have reported volume reductions in the frontal and temporal regions as neural markers for psychosis [8]. MRIs were obtained from 20 patients with MAP and 20 demographically matched HCs to assess for differences in regional brain volume using voxel-based morphometry (VBM) [9]. The VBM analyses showed significant gray matter volume reductions in the left perisylvian structures and white matter volume reduction in the orbitofrontal area in subjects with MAP compared with HCs. The volume reductions in the left perisylvian structure suggested a pathophysiologic similarity to schizophrenia.

In chronic MA users, the risk of MAP is five times higher while using as compared to during abstinence [10]. Studies have also reported that one-third of these patients with MAP have extended periods of inpatient stay lasting more than 60-90 days [11]. Although the psychopathology of MAP is distinct and different from schizophrenia, antipsychotics remain the mainstay for the treatment of psychotic symptoms. Chronic MA users develop significant CNS neurotoxicity at a faster rate [12], as revealed by animal studies and neuroimaging studies, and this could be a plausible reason behind their guarded response to antipsychotic agents. The lower baseline striatal dopamine 2 receptor (D2R) availability and dopamine release in MA abusers [13] could be another reason for poor response to treatment. Few trials have suggested the superior efficacy of olanzapine and quetiapine over haloperidol [14]. The risk of seizures and movement disorders has also been reported due to the interaction between haloperidol and MA, leading to toxicity of GABAergic neurons [15]. Therefore, the slow titration of clozapine in refractory MAP remains an appropriate treatment option, and given the risk of seizures, a prophylactic addition of valproic acid or other agents like lamotrigine, gabapentin, and topiramate may be considered [16].

There are a few reports that recommend the use of benzodiazepines [17]; however, caution should be exercised due to the risk of significant disinhibition, and altered sleep patterns may further worsen the course. Amantadine acts via antagonism of the NMDA receptor. It decreases the toxic effects of the glutamatergic neurotransmitter system, which plays an important role in many psychiatric disorders [18]. There have been many trials involving memantine (NMDA receptor antagonist similar to amantadine) as a drug for adjunctive use with antipsychotics for schizophrenia [19]. A meta-analysis of these trials indicated that memantine led to improvement in cognitive functions when used as an adjunctive agent in schizophrenia [20]. To the best of our knowledge, there are no reports about the use of amantadine for MAP. The possible explanation of its utility is supported by the evidence of its ability to modulate the glutamatergic system, the fact that it possesses neuroprotective activity, and its role in improving cognitive deficits and memory. Table 2 provides a few psychopharmacological approaches for clinical consideration. 

**Table 2:** Summary of evidence-based treatment strategies for MAP NMDA: N-methyl-D-aspartate

Treatment strategies for methamphetamine-related psychosis (MAP)
Two trials have suggested that quetiapine and olanzapine are superior to haloperidol
A few case reports support the use of clozapine as an effective alternative in refractory cases
Benzodiazepines may be used but caution should be exercised due to the risk of disinhibition and respiratory depression due to concomitant opioid use
NMDA antagonists have been used as adjunctive agents for schizophrenia with a modest response
Prophylactic addition of antiepileptics in patients with risk of seizure must be considered
Refractory cases (about 33%) may take a longer time to respond, which likely extends the length of stay in inpatient units

## Conclusions

The risk of psychosis among chronic MA users is well known. With the recent enforcement of opioid-related legislation and policies, there has been a shift in trends and patterns of MA use. The current MAP presentations are more atypical given the use of a polysubstance and the wider availability of counterfeit prescriptions with unknown compounds. There is a substantial proportion of patients with MAP who would not respond to conventional treatment, and the early use of clozapine may yield better outcomes in such cases. There is a need for more clear consensus guidelines to comprehensively assess and treat both acute and long-term mental health effects of MA use. Amantadine has been demonstrated to have efficacy in improving cognitive function in refractory schizophrenia and could be used in select patients due to its unique psychopharmacologic actions. Further research and proactive measures are required to address a potential new epidemic in the making.

## References

[1] Luckower T (2007). Follow-up on the Combat Methamphetamine Epidemic Act of 2005: review of oral decongestants. J Am Pharm Assoc (2003).

[2] Arunogiri S, Foulds JA, McKetin R, Lubman DI (2018). A systematic review of risk factors for methamphetamine-associated psychosis. Aust N Z J Psychiatry.

[3] Courtney KE, Ray LA (2016). Clinical neuroscience of amphetamine-type stimulants: from basic science to treatment development. Prog Brain Res.

[4] Kamei H, Nagai T, Nakano H (2006). Repeated methamphetamine treatment impairs recognition memory through a failure of novelty-induced ERK1/2 activation in the prefrontal cortex of mice. Biol Psychiatry.

[5] Hsieh JH, Stein DJ, Howells FM (2014). The neurobiology of methamphetamine induced psychosis. Front Hum Neurosci.

[6] Chen CK, Lin SK, Sham PC (2003). Pre-morbid characteristics and co-morbidity of methamphetamine users with and without psychosis. Psychol Med.

[7] Vuletic D, Dupont P, Robertson F, Warwick J, Zeevaart JR, Stein DJ (2018). Methamphetamine dependence with and without psychotic symptoms: a multi-modal brain imaging study. Neuroimage Clin.

[8] Courtney KE, Ray LA (2014). Methamphetamine: an update on epidemiology, pharmacology, clinical phenomenology, and treatment literature. Drug Alcohol Depend.

[9] Aoki Y, Orikabe L, Takayanagi Y (2013). Volume reductions in frontopolar and left perisylvian cortices in methamphetamine induced psychosis. Schizophr Res.

[10] McKetin R, Hickey K, Devlin K, Lawrence K (2010). The risk of psychotic symptoms associated with recreational methamphetamine use. Drug Alcohol Rev.

[11] Iwanami A, Sugiyama A, Kuroki N (1994). Patients with methamphetamine psychosis admitted to a psychiatric hospital in Japan. A preliminary report. Acta Psychiatr Scand.

[12] Berman S, O'Neill J, Fears S, Bartzokis G, London ED (2008). Abuse of amphetamines and structural abnormalities in the brain. Ann N Y Acad Sci.

[13] Wang GJ, Smith L, Volkow ND (2012). Decreased dopamine activity predicts relapse in methamphetamine abusers. Mol Psychiatry.

[14] Verachai V, Rukngan W, Chawanakrasaesin K (2014). Treatment of methamphetamine-induced psychosis: a double-blind randomized controlled trial comparing haloperidol and quetiapine. Psychopharmacology (Berl).

[15] Hatzipetros T, Raudensky JG, Soghomonian JJ, Yamamoto BK (2007). Haloperidol treatment after high-dose methamphetamine administration is excitotoxic to GABA cells in the substantia nigra pars reticulata. J Neurosci.

[16] Williams AM, Park SH (2015). Seizure associated with clozapine: incidence, etiology, and management. CNS Drugs.

[17] Shoptaw SJ, Kao U, Ling W (2009). Treatment for amphetamine psychosis. Cochrane Database Syst Rev.

[18] Danysz W, Dekundy A, Scheschonka A, Riederer P (2021). Amantadine: reappraisal of the timeless diamond-target updates and novel therapeutic potentials. J Neural Transm (Vienna).

[19] de Lucena D, Fernandes BS, Berk M (2009). Improvement of negative and positive symptoms in treatment-refractory schizophrenia: a double-blind, randomized, placebo-controlled trial with memantine as add-on therapy to clozapine. J Clin Psychiatry.

[20] Kishi T, Iwata N (2013). NMDA receptor antagonists interventions in schizophrenia: meta-analysis of randomized, placebo-controlled trials. J Psychiatr Res.

